# Association of adherence to the enhanced recovery after surgery pathway and outcomes after laparoscopic total gastrectomy

**DOI:** 10.1186/s12871-024-02433-9

**Published:** 2024-03-22

**Authors:** Yiming Hao, Qingchuan Zhao, Kun Jiang, Xiangying Feng, Yumei Ma, Jianzhong Zhang, Xi’an Han, Gang Ji, Hailong Dong, Huang Nie

**Affiliations:** 1 Department of Gastrointestinal Surgery, Xijing Hospital, Fourth Military Medical University, Xi’an, China; 2 Department of Anesthesiology, Xijing Hospital, Fourth Military Medical University, Xi’an, China; 3 Department of Digital Center, Xijing Hospital, Fourth Military Medical University, Xi’an, China; 4 The Unimed Scientific Inc, Wu Xi, China

**Keywords:** Laparoscopic total gastrectomy, Enhanced recovery after surgery, Compliance, Complications, Optimal postoperative recovery

## Abstract

**Objective:**

The current study used a composite outcome to investigate whether applying the ERAS protocol would enhance the recovery of patients undergoing laparoscopic total gastrectomy (LTG).

**Exposures:**

Laparoscopic total gastrectomy and perioperative interventions were the exposure. An ERAS clinical pathway consisting of 14 items was implemented and assessed. Patients were divided into either ERAS-compliant or non-ERAS-compliant group according the adherence above 9/14 or not.

**Main outcomes and measures:**

The primary study outcome was a composite outcome called ‘optimal postoperative recovery’ with the definition as below: discharge within 6 days with no sever complications and no unplanned re-operation or readmission within 30 days postoperatively. Univariate logistic regression analysis and multivariate logistic regression analysis were used to model optimal postoperative recovery and compliance, adjusting for patient-related and disease-related characteristics.

**Results:**

A total of 252 patients were included in this retrospective study, 129 in the ERAS compliant group and 123 in the non-ERAS-compliant group. Of these, 79.07% of the patients in ERAS compliant group achieved optimal postoperative recovery, whereas 61.79% of patients in non-ERAS-compliant group did (*P* = 0.0026). The incidence of sever complications was lower in the ERAS-compliant group (1.55% vs. 6.5%, *P* = 0.0441). No patients in ERAS compliant group had unplanned re-operation, whereas 5.69% (7/123) of patients in non-ERAS-compliant group had (*p* = 0.006). The median length of the postoperative hospital stay was shorter in the in the ERAS compliant group (5.51 vs. 5.68 days, *P* = 0.01). Both logistic (OR 2.01, 95% CI 1.21–3.34) and stepwise regression (OR 2.07, 95% CI 1.25–3.41) analysis showed that high overall compliance with the ERAS protocol facilitated optimal recovery in such patients. In bivariate analysis of compliance for patients who had an optimal postoperative recovery, carbohydrate drinks (*p* = 0.0196), early oral feeding (*P* = 0.0043), early mobilization (*P* = 0.0340), and restrictive intravenous fluid administration (*P* < 0.0001) were significantly associated with optimal postoperative recovery.

**Conclusions and relevance:**

Patients with higher ERAS compliance (almost 70% of the accomplishment) suffered less severe postoperative complications and were more likely to achieve optimal postoperative recovery.

## Introduction

Gastric cancer was the third leading cause of cancer-related death worldwide and over 1 million new cases occurred globally in 2018 [1]. Surgery remains the mainstay of gastric cancer treatment. Despite recent advances in surgical techniques such as minimally invasive surgery, the postoperative complication incidence reached up as high as 30% [2–4] and mortality up to 4% [5]. Perioperative complications have been shown to be strongly associated with poor long-term outcomes in very large surgical series [6]. It is not surprising that any efforts which could decrease the complications would be of interests to the surgeons and perioperative health providers.

Over the past 20 years, the Enhanced Recovery After Surgery (ERAS) pathway, a multidisciplinary approach to reduce perioperative stress has been reported to improve the recovery quality after surgery in many surgical specialties by reducing complications and shortening length of hospital stay [7–11]. Recommendations were developed for enhanced recovery items covering topics involved of preadmission, admission, intraoperative care and postoperative care. Several key recommendations about preoperative nutrition, smoking cessation, multimodal analgesia, postoperative fluid optimization and early mobilization have been demonstrated to improve outcomes after surgery [12–16]. Notably however, its safety and efficacy in gastric cancer patients especially those undergoing total gastrectomy warrants further dedicated research [17–19].

Recently, 3 prospective trials from Japan (JCOG1401 trial), Korea (KLASS03 trial) and China (CLASS02 trial) respectively demonstrated that laparoscopic total gastrectomy (LTG) could be safely performed as open total gastrectomy for gastric cancer patients by experienced surgeons. Yet few studies have been done to verify the effect of implementing ERAS pathway on patients’ postoperative recovery after LTG. We wonder whether application of ERAS pathway is feasible in patients undergoing LTG and whether ERAS adherence is associated with the recovery quality. The current cohort study was conducted to investigate the effects of ERAS pathway compliance, patient characteristics, and surgical factors on patient outcomes after LTG.

## Materials and methods

### Patients and study design

This retrospective cohort study enrolled consecutive patients from our hospital between 18 July 2017 and 31 May 2020. Patients were deemed eligible for inclusion if they were above 18 years old and scheduled for elective laparoscopic total gastrectomy. We excluded those who had synchronous or metachronous malignant tumors in other organs within the past 5 years, a history of any gastric surgery.

### Ethics approval and consent to participate

The Medical Ethics Committee of First Affiliated Hospital of Fourth Military Medical University reviewed and approved (ID code KY20172041-1) the protocol of this study and exempted the requirement for obtaining informed consent due to the retrospective, minimal-risk nature of the study. The study was registered in the Chinese Clinical Trial Registry(ChiCTR-ONC-17,012,230).

### Perioperative treatment

All the surgeons made an agreement on surgical technical details and performed enough cases of laparocopic subtotal or total gastrectomy. A standardized clinical pathway consisting of 14 components was applied peri-operatively (Table 1). The discharge criteria included a normal body temperature, tolerating to soft diet, good pain management with oral analgesics, no need for intravenous fluids and independent mobilization [20].

**Table 1:** Indicators used to assess compliance with ERAS pathways

Care pathways	Measured ERAS recommendations
Preoperative	1) Preadmission patient education: Preoperative pulmonary function training is required before admitted to the hospital. 2) Preoperative nutrition support: Patients with NRS2002 > = 3 are given enteral nutrition or parenteral support before surgery. 3) Tobacco smoking and alcohol cessation: >3 weeks tobacco smoking cessation; >4 weeks alcohol cessation. 4) Preoperative carbohydrate loading: Take 200 ml carbohydrate-loading drinks 2 h before surgery. 5) Bowel preparation: Without bowel preparation.
Intraoperative	6) Maintenance of intraoperative normothermia: Use cutaneous warming to keep deep body temperature 36 centigrade above. 7) Multimodal analgesia: Use incisional infiltration with local anesthetics and NSAIDs i.v. prior to skin incision. 8) PONV prophylaxis: Patients with two risk factors should be given prophylaxis with dexamethasone upon induction or a serotonin receptor antagonist at the end of surgery. 9) Antibiotic prophylaxis: Given antibiotic prophylaxis before skin incision.
Postoperative	10) Early oral feeding: Start clear fluids or liquid nutrition at POD 1. 11) Early mobilization: Siting in chair at POD 0 and ambulation at POD 1. 12) Avoidance or early removal of nasogastric tube: If placed, removed it in the morning of POD 1. 13) Avoidance or early removal of urinary catheter: If placed, removed it at POD 1. 14) Restrictive intravenous fluid administration: End of intravenous fluid within POD 5.

*NRS2002* nutritional risk screening 2002, *PONV* postoperative nausea and vomiting, *NSAIDs* non-steroidal anti-inflammatory durgs, *i.v.* intravenous injection, *POD0* postoperative day 0, *POD1* postoperative day 1

### Outcomes and definitions

Data pertaining to baseline demographics, compliance with ERAS protocol, clinical outcomes, postoperative complications, mortality, length of postoperative hospital stay, and 30-day postoperative readmission were obtained prospectively. Data were collected in two ways. The electronic medical record system including ERAS-structured medical records was used to source data relating to complications, ERAS protocol compliance, and other objective data such as laboratory test results and length of postoperative hospital stay. Subjective data such as pre-rehabilitation and postoperative mobilization duration were self-reported by the patients and recorded via a bedside electronic device. All the data were extracted automatically and saved in a database prior to analysis. The system we used for data collection and derivation was developed by the Unimed Scientific Inc. (Wu Xi, China).

Compliance with ERAS protocol was measured for each component of the program (Table 1). Patients with ERAS complaint were considered as more than 9 ERAS recommendations as outlined in Table 1 were met (any 10 out of 14) [21]. Thirty-day readmission was defined as readmission within 30 days after the surgery (admission for chemotherapy was excluded). Optimal postoperative recovery was defined as discharge within 6 days after surgery with no severe complications (severe complications refers to those classified as Clavien–Dindo grade III or higher [21]), no unplanned re-operation and no 30-day readmission after surgery [22].

### Statistical analysis

Data were summarized as means and standard deviations or median and inter-quartile range for continuous variables and frequencies and proportions for categorical variables. Between-group differences were assessed via the two-tailed Student’s *t*-test (for parametric variables) or the Mann-Whitney U test (for non-parametric variables). Categorical variables were analyzed via the chi-square test, CMH-chi-square test, or Fisher’s exact test as appropriate. Univariate logistic regression analysis and multivariate logistic regression analysis (stepwise regression method) were used to model optimal postoperative recovery and compliance, adjusting for patient-related and disease-related characteristics. Odds ratios (ORs) and 95% confidence intervals (CIs) were calculated to assess differences between patients with ERAS-compliant courses and those with ERAS-non-compliant courses. Statistical tests were interpreted at a two-sided significance level of 5%. All statistical analyses were performed using SAS version 9.3 (SAS Institute Inc., Cary, NC, USA).

## Results

### Patient characteristics and overall postoperative outcomes

**Fig. 1:**
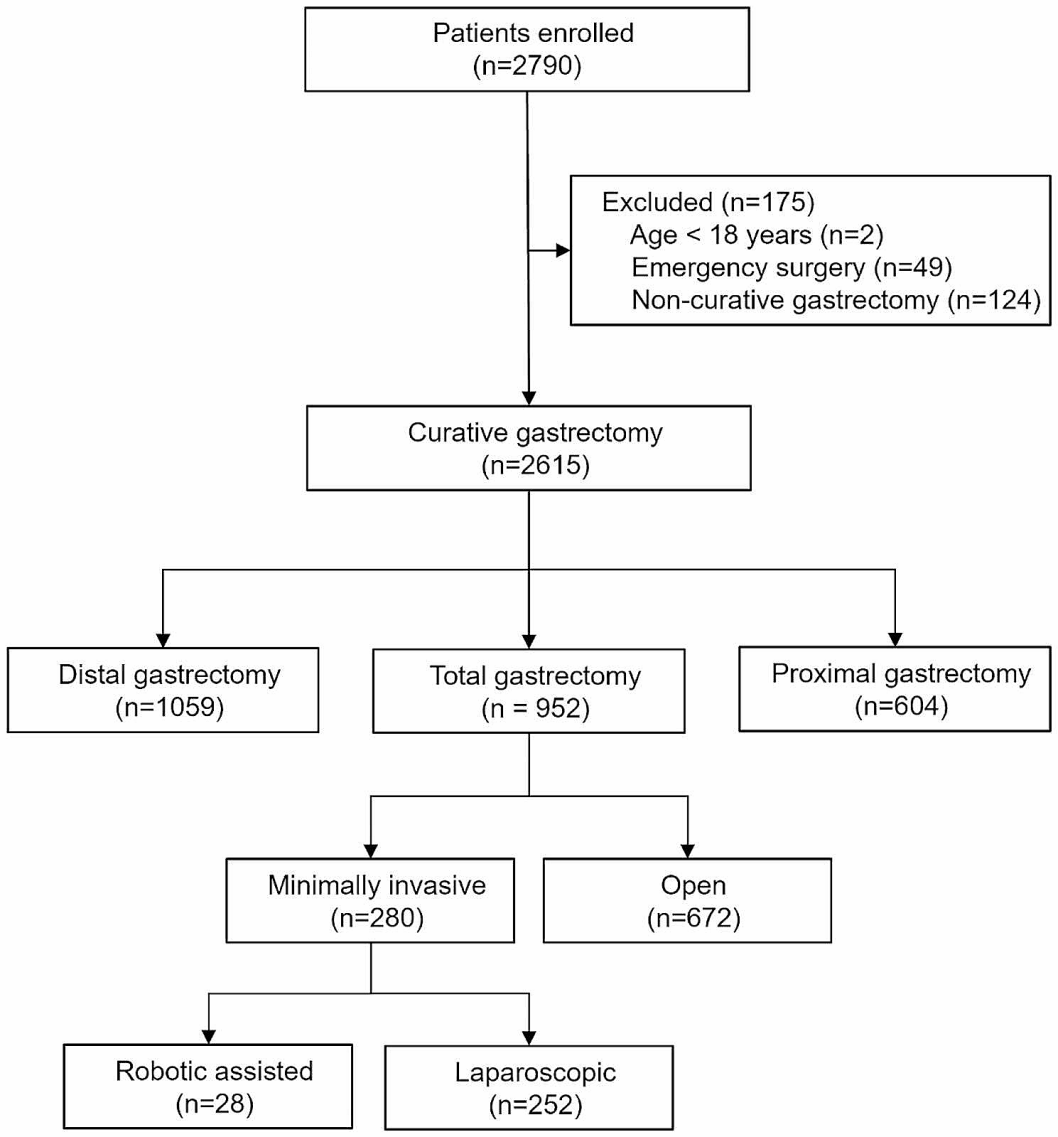
Flow chart of patients included in the study

From 18 July 2017 to 31 May 2020, 2790 patients received gastrectomy in our hospital. 175 cases were excluded by age less than 18, emergency surgery or non-curative gastrectomy. Among the 2615 curative gastrectomies, 252 were lasparoscopical total gastrectomies. Therefore, a total of 252 patients were enrolled in the study (Fig. 1). The mean age was 60.07 years, 82.94% were male, and 12.17% were ASA ≥ 3. The ERAS compliant and ERAS Non-compliant groups showed no difference in patient demographics (Table 2).

**Table 2:** Patient characteristics

		Overall *n* = 252	ERAS Compliant (ERAS Pathways > = 10) *n* = 129	ERAS Non-compliant (ERAS Pathways < 10) *n* = 123	P Value
Age	Mean ± SD	60.07 ± 9.75	60.64 ± 9.14(*n* = 129)	59.46 ± 10.36(*n* = 123)	0.2345
Male	N(%)	209(82.94%)	106(82.17%)(*n* = 129)	103(83.74%)(*n* = 123)	0.7406
BMI	Mean ± SD	23.38 ± 3.34	23.39 ± 3.32(*n* = 123)	23.37 ± 3.39(*n* = 120)	0.9576
Anemia	N(%)	88(35.06%)	45(35.16%)(*n* = 128)	43(34.96%)(*n* = 123)	0.9739
Abnormal Creatinine	N(%)	22(8.80%)	11(8.59%)(*n* = 128)	11(9.02%)(*n* = 122)	0.9061
Comorbidity	N(%)	45(17.86%)	27(20.93%)(*n* = 129)	18(14.63%)(*n* = 123)	0.1921
ASA ≥ 3	N(%)	28(12.17%)	12(10.26%)(*n* = 117)	16(14.16%)(*n* = 112)	0.3655
Duration of surgery, min	Mean ± SD	272.70 ± 61.12	274.92 ± 59.51(*n* = 129)	270.37 ± 62.93(*n* = 123)	0.5559
Blood loss, mL	median (IQR)	100(50)	100(50)(*n* = 129)	100(100)(*n* = 123)	0.5135
Intraoperative transfusion (Yes)	N(%)	19(7.54%)	8(6.20%)(*n* = 129)	11(8.94%)(*n* = 123)	0.4100
Pathologic TNM stage					0.8760
I A	N(%)	31(12.30%)	18(13.95%)	13(10.57%)	
I B	N(%)	24(9.52%)	12(9.30%)	12(9.76%)	
II A	N(%)	41(16.27%)	23(17.83%)	18(14.63%)	
II B	N(%)	36(14.29%)	15(11.63%)	21(17.07%)	
III A	N(%)	53(21.03%)	27(20.93%)	26(21.14%)	
III B	N(%)	50(19.84%)	26(20.16%)	24(19.51%)	
III C	N(%)	17(6.75%)	8(6.20%)	9(7.32%)	

*ERAS* enhanced recovery after surgery, *BMI* body mass index

For patients in the study, the mean operative duration was 4.54 h. The incidence of severe postoperative complications was 3.97% and no mortality was reported (Table 3). Optimal postoperative recovery as defined above in the methods section was achieved in 70.63% of patients. The median length of postoperative hospital stay was 5.54 days and the 30-day readmission rate was 1.59%. The re-operation rate was 2.78%.

**Table 3:** Postoperative outcomes

		Overall *n* = 252	ERAS Compliant (ERAS Pathways ≥ 10) *n* = 129	ERAS Non-compliant (ERAS Pathways < 10) *n* = 123	P Value
Optimal postoperative recovery	N(%)	178(70.63%)	102(79.07%)	76(61.79%)	0.0026
Postoperative hospital stay	median (IQR)	5.54(1.90)	5.51(1.09)	5.68(1.95)	0.0100
Severe complications	N(%)	10(3.97%)	2(1.55%)	8(6.50%)	0.0441
Unplanned Re-operation	N(%)	7(2.78%)	0(0.00%)	7(5.69%)	0.0060
30 day Readmission	N(%)	4(1.59%)	1(0.78%)	3(2.44%)	0.2908

*ERAS* enhanced recovery after surgery

### Patients in ERAS compliant group suffered less severe complications and had higher optimal postoperative recovery ratio

According to the definition, 129 patients were included in ERAS compliant group and 123 patients in ERAS Non-compliant group. A total of 1.55% (2/129) of patients in ERAS compliant group had severe complications, whereas the ratio was 6.50% in ERAS Non-compliant group (*p* = 0.0441). 79.07% (102/129) of patients in ERAS compliant group achieved optimal postoperative recovery, whereas the ratio for ERAS Non-compliant group was 61.79% (*p* = 0.0026). The median length of the postoperative hospital stay was 5.51 days in the ERAS compliant group and 5.68 days in the ERAS Non-compliant group (*p* = 0.01). No patient in ERAS compliant group had unplanned re-operation, 5.69% (7/123) of patients in ERAS Non-compliant group had re-operation (*p* = 0.006). There were no significant differences between the two groups with regard to readmission rates (0.78% vs. 2.44%) (Table 3).

### Compliance with ERAS pathways was related to optimal postoperative recovery

In our study, the results of univarial analysis indicated that preoperative carbohydrate drinks (*p* = 0.0196), early oral feeding (*p* = 0.0043), early mobilization (*p* = 0.0340), and restrictive intravenous fluid administration (*p* < 0.0001) were significantly associated with optimal postoperative recovery (Table 4). In multivariate logistic regression analysis, with regard to patient and intervention factors, only ERAS pathways compliance (OR 2.01, 95% CI 1.21–3.34) was significantly associated with optimal postoperative recovery (Table 5). The result showed the same conclusion when using stepwise regression analysis (OR 2.07, 95% CI 1.25–3.41) (Table 6).

**Table 4:** Compliance with ERAS pathways and bivariate analysis of compliance for patients who had an optimal postoperative recovery

ERAS Recommendations	Compliance in Total Cohort *n* = 252	Compliance in Optimal Postoperative Recovery Cohort *n* = 178	Compliance in Non-optimal Postoperative Recovery Cohort *n* = 74	P Value
Preadmission patient education (*n* = 188)	123(65.43%)	91(68.42%)	32(58.18%)	0.1793
Preoperative nutrition support (*n* = 252)	245(97.22%)	175(98.31%)	70(94.59%)	0.1017
Tobacco smoking and alcohol cessation (*n* = 190)	114(60.00%)	86(63.70%)	28(50.91%)	0.1025
Preoperative carbohydrate loading (*n* = 252)	33(13.10%)	29(16.29%)	4(5.41%)	0.0196
Bowel preparation (*n* = 252)	200(79.37%)	145(81.46%)	55(74.32%)	0.2023
Maintenance of intraoperative normothermia (*n* = 252)	147(58.33%)	102(57.30%)	45(60.81%)	0.6070
Multimodal analgesia (*n* = 252)	121(48.02%)	83(46.63%)	38(51.35%)	0.4944
PONV prophylaxis (*n* = 252)	237(94.05%)	168(94.38%)	69(93.24%)	0.7279
Antibiotic prophylaxis (*n* = 252)	252(100%)	178(100%)	74(100%)	1.0000
Early oral feeding (*n* = 172)	35(20.35%)	32(25.81%)	3(6.25%)	0.0043
Early mobilization (*n* = 205)	189(92.20%)	141(94.63%)	48(85.71%)	0.0340
Avoidance or early removal of nasogastric tube (n-252)	197(78.17%)	142(79.78%)	55(74.32%)	0.3400
Avoidance or early removal of urinary catheter (n-252)	250(99.21%)	176(98.88%)	74(100.00%)	0.3599
Restrictive intravenous fluid administration (n-252)	233(92.46%)	178(100.00%)	55(74.32%)	< 0.0001

*ERAS* enhanced recovery after surgery, *PONV* postoperative nausea and vomiting

**Table 5:** Univariable and multivariable analysis of predictor factors for optimal postoperative recovery

Factors	Univariable		Multivariable	
	OR (95% CI)	P Value	OR (95% CI)	P Value
Age (< 65 vs. ≥ 65)	0.73[0.45–1.18]	0.2847	0.74[0.42–1.29]	0.3717
Sex (Male vs. Female)	1.05[0.58–1.92]	0.8902	1.47[0.76–2.84]	0.3322
BMI(≥ 30/[25,30)/[18.5,25) vs. < 18.5)	0.84[0.58–1.20]	0.4189	0.74[0.49–1.13]	0.2415
Anemia (Yes vs. No)	1.00[0.62–1.60]	0.9871	1.09[0.64–1.86]	0.7813
ASA (≥ 3 vs. < 3)	0.69[0.34–1.39]	0.3828	0.56[0.26–1.21]	0.2152
Creatinine (Normal vs. Abnormal)	0.69[0.29–1.65]	0.4866	0.84[0.34–2.11]	0.7604
Operation duration(≤ 4 h vs. > 4 h)	0.96[0.59–1.56]	0.8796	0.71[0.40–1.28]	0.3387
ERAS Pathways Compliance (ERAS Pathways ≥ 10 vs. ERAS Pathways < 10)	2.34[1.46–3.73]	0.0029	2.01[1.21–3.34]	0.0238

*BMI* body mass index, *ASA* american society of anesthesiologists, *ERAS* enhanced recovery after surgery

**Table 6:** Univariable and multivariable analysis of predictor factors for optimal postoperative recovery (stepwise regression method)

Factors	OR (95% CI)	P value
ERAS Pathways Compliance (ERAS Pathways ≥ 10 vs. ERAS Pathways < 10)	2.07[1.25–3.41]	0.0172

*ERAS* enhanced recovery after surgery

## Discussion

The present study investigated the effects of ERAS compliance on patient recovery after LTG. The primary outcome in our study was a composite outcome called ‘optimal postoperative recovery’, including discharge within 6 days postoperatively, no severe complications, no unplanned re-operation, and no 30-day readmission. This primary outcome referring to the essence of the enhanced recovery after surgery, showed more patients-outcome consideration when compared with the usually used outcomes such as hospital stay, since a shorter hospital stay alone does not always guarantee the high recovery quality.

In our study, 79.07% of patients in the ERAS compliant group achieved optimal postoperative recovery, whereas only 61.79% of patients in the ERAS non-compliant group met the same criteria. The ERAS compliant group showed lower sever postoperative complication incidence and re-operation rates. Meanwhile, the median length of the postoperative hospital stay was shorter in ERAS compliant group. In multivariate logistic regression analysis, only ERAS pathways compliance was significantly associated with optimal postoperative recovery. These results indicated that higher ERAS adherence facilitated optimal postoperative recovery after LTG. We take 10 out of 14 ERAS intervention adherence as ERAS compliant criteria for two reasons. First, most ERAS studies found that about 70% adherence to ERAS program could improve the clinical outcomes [23]. The incidence of severe complications (Clavien–Dindo grade III or higher) was 3.82% in the current study. The result is similar to those of Tanaka et al.’s [24] study in which 19.2% of complications were grade II or higher and 4.1% were grade III or higher in the ERAS group.

Studies showed that there was a relationship between compliance and complications [11, 25]. The study indicates that the ERAS protocol can be safely implemented in laparoscopic total gastrectomy. Nowadays, the length of hospital stay ranges from 4.7 days to 8 days in gastrectomy studies [26–28]. Indeed, implementing ERAS pathway can reduce the hospital stay in gastrointestinal cancer surgery. A major concern of ERAS is earlier hospital discharge may result in increased readmission [10]. There were no 30-day deaths, and the unplanned re-operation rates were 1.59% in the study. 30-day readmission rate was not increased in the ERAS compliant group compared with the ERAS non-compliant group (4.28% vs. 4.56%, *P* = 0.7750).

The current study has external generalizability. The patients it included were consecutive patients who had elective gastrectomies from 2017 to 2020. Now, gastric cancer patients enrolled in most ERAS studies were early stage (stage I), good performance status (ECOG 0–1) and ASA ≤ 3 [24, 28–30]. We did not exclude patients based on characteristics such as age, body mass index, anemia, comorbidity, previous abdominal surgery, ASA score, gastrectomy procedure, pathology stage, or other factors, so the conclusions can be reasonably generalized to the general gastric cancer patients.

There were several limitations to the present study. First, the study was performed at a single center and focused in the laproscopic total gastrectomy patients, which may limit the generalizability. Second, causal associations can only be inferred, because the investigation was an retrospective study and only known potentially confounding variables were controlled for. Lastly, the limitation includes missing data with respect to compliance judgment with the four ERAS recommendations (preoperative pulmonary rehabilitation, preoperative smoking and alcohol consumption, early oral feeding, and early mobilization). These data were reported by the patients and recorded via a bedside electronic device. In the early period of the study, patients were not thoroughly supervised to ensure that every patient reported the data, though this situation was improved in the latter part of the study. These missing data may have resulted in bias or imprecision.

## Conclusions

In conclusion, this study shows that an increase in ERAS protocol compliance is associated with better short-term clinical outcomes in LTG. Therefore, auditing of adherence to ERAS is essential to patient postoperative outcomes. In the future, the feasibility of ERAS program for gastrectomy in general population can be investigated by the multi-center study with patient collection.

## Data Availability

The datasets generated during and/or analyzed during the current study are not publicly available due to statutory provisions regarding data and privacy protection. Huang Nie should be contacted if someone wants to request the data from this study.

## Declarations

### Trial registry number

This research was registered in the Chinese Clinical Trial Registry (ChiCTR) (ID code ChiCTR-ONC-17012230) on August 2nd, 2017.
